# Comparison of pattern electroretinograms of glaucoma patients with parafoveal scotoma versus peripheral nasal step

**DOI:** 10.1038/s41598-019-39948-y

**Published:** 2019-03-05

**Authors:** Kyoung In Jung, Sooji Jeon, Yong Chan Kim, Chan Kee Park

**Affiliations:** 1 Department of Ophthalmology, Seoul St. Mary’s Hospital, College of Medicine, The Catholic University of Korea, Seoul, Republic of Korea; 2 Department of Ophthalmology, Hallym University College of Medicine, Chuncheon Sacred Heart Hospital, Chuncheon, Republic of Korea

## Abstract

Retinal ganglion cells are distributed disproportionately with retinal eccentricity. Pattern electroretinogram (PERG) stimuli resulted in reduced responses with more eccentric stimuli. Therefore, we investigated whether PERG amplitude is associated with the location of visual field (VF) defect in primary open-angle glaucoma. Data from Twenty-nine glaucoma patients with a parafoveal scotoma (PFS) within the central 10° of fixation, 23 glaucoma patients with a peripheral nasal step (PNS), and 27 normal control subjects were analyzed in this study. Electroretinograms (ERGs) were obtained using a commercial ERG stimulator (Neuro-ERG). The thickness of the ganglion cell-inner plexiform layer (GCIPL) was measured using spectral-domain optical coherence tomography. A lower N95 amplitude was observed in both PFS and PNS compared to the normal control (Both P < 0.001). The N95 amplitude of the PFS group was significantly lower than that of the PNS group (P = 0.034). Average GCIPL thickness correlated positively with N95 amplitude (r = 0.368, P = 0.002), but did not correlate significantly with global mean sensitivity (r = 0.228, P = 0.073) or mean deviation on 24-2 standard automated perimetry (r = 0.173, P = 0.176). In conclusion, parafoveal VF defects were associated with the lower PERG amplitude. Therefore, it is necessary to take into account the location of VF defects in evaluating PERGs of glaucoma patients.

## Introduction

The visual field (VF) test has been the foundation of glaucoma diagnosis and treatment^1^. However, VF tests are influenced by fatigue or learning effects, and depend on subjective patient responses^2^. Objective functional tests, such as the pattern electroretinogram (PERG), attempt to address this inherent variability, although they have not yet been established as a routine examination^1^.

The PERG, which measures responses to a phase-reversing pattern, was found to directly reflect the function of retinal ganglion cells (RGCs), at least from the inner retina^3^. The PERG has yielded encouraging results in differentiating glaucoma patients from normal subjects^2–6^. It can be helpful in the early detection of glaucomatous damage, as abnormal PERGs have been obtained from patients with ocular hypertension; the PERG can foresee, to some extent, who will present VF loss^3,7,8^.

PERGs are known to generally measure electrical activity upon central stimulation of the macula (typically <15° stimulus field)^3,9^. Recently, several types of PERG were developed in which the visual angle was extended to 44° horizontally, which is similar to the horizontal visual angle (52°) examined by 24-2 standard automated perimetry (SAP 24-2)^5,10^. It has not been extensively studied whether PERG parameters differ depending on the location of VF defects in glaucoma patients, even though conventional PERGs have been regarded as a central vision test stimulating the macula.

RGCs are distributed disproportionately, more densely populating the paracentral retina than the peripheral retina^11^. For example, over 30% of RGCs are located in the central 10° of the VF^12^. Early RGC damage can occur in the paracentral retina, even in glaucoma patients with normal SAP 24-2 because only 4 points of the 24-2 SAP are located within the central 10°^13^. The PERG response with stimulation of more eccentric regions decreased even at their respective optimal spatial frequencies^14^.

Given those findings, a PERG response may differ with subjects with parafoveal scotoma (PFS) compared to those with peripheral nasal step (PNS) quantitatively and qualitatively, even with the extended visual angle. Therefore, we compared the PERG results between glaucoma patients with PNS and PFS.

## Methods

### Subjects

This cross-sectional study was approved by the Institutional Review Board of the Catholic University of Korea, Seoul, Korea, and followed the tenets of the Declaration of Helsinki. Informed consent was obtained from all the patients. Glaucoma patients who met the inclusion criteria were consecutively included from all patients the glaucoma clinic of Seoul St. Mary’s Hospital between October 2017 and November 2017. Inclusion criteria were: best-corrected visual acuity ≥20/40, axial length <27 mm, and an open angle. Patients with diseases that might affect the parapapillary or macular areas, with neurologic diseases, history of glaucoma surgery, or with unreliable VF tests, were excluded. When both eyes met the inclusion criteria, one eye per individual was randomly selected for the study.

### Measurements

All patients underwent a complete ophthalmic examination, including slit-lamp biomicroscopy, Goldmann applanation tonometry, gonioscopy, axial length measurement, central corneal thickness measurement, and dilated fundus biomicroscopy. Stereoscopic optic disc photography was also performed on all subjects.

A glaucomatous VF defect was defined as a cluster of 3 or more points with a *P*-value < 5%, one of which had a *P*-value of < 1% for the pattern deviation plot. Eyes showing a glaucomatous optic disc, such as diffuse or focal rim thinning, notching, or retinal nerve fiber layer defect with corresponding glaucomatous VF damage, fulfilled the criteria for PFS or PNS.

#### Optical Coherence Tomography

Spectral-domain optical coherence tomography (OCT) imaging was performed using Cirrus HD-OCT version 6.0 (Carl Zeiss Meditec, Inc.). Ganglion cell-inner plexiform layer (GCIPL) thickness was measured using a macular cube scan. The protocol for GCIPL thickness has been previously described in detail^15,16^. Ganglion cell analysis software was used to measure the average, minimum, and sectoral (superior, superotemporal, superonasal, inferior, inferotemporal, and inferonasal) GCIPL thicknesses in a 14.13 mm^2^ elliptical annulus with vertical inner and outer radii of 0.5 and 2.0 mm, respectively, and horizontal inner and outer radii of 0.6 and 2.4 mm, respectively. Retinal nerve fiber layer (RNFL) thickness was determined using the optic Disc Cube 200 × 200 scan mode. Optic disc parameters such as disc area, rim area, average cup-to-disc ratio (CDR), vertical CDR, and cup volume were analyzed. Poor-quality images with signal strength <6 were discarded.

#### Visual Field Testing

All patients underwent SAP 24-2 with a Humphrey field analyzer (Carl Zeiss Meditec, Dublin, CA). Goldmann size III targets were used with the Swedish interactive threshold algorithm (SITA) standard program. The mean deviation (MD) and pattern standard deviation (PSD) were evaluated. Mean sensitivity (MS) was evaluated on threshold printout in VF tests. VF sensitivity was evaluated using the logarithmic decibel (dB) [10 × log(1/Lambert)] scale.

Reliable tests were defined as those with <15% fixation losses, false positives, or false negatives. A second VF test was conducted if the first one was not reliable.

### VF Criteria for PFS and PNS

**Figure 1:**
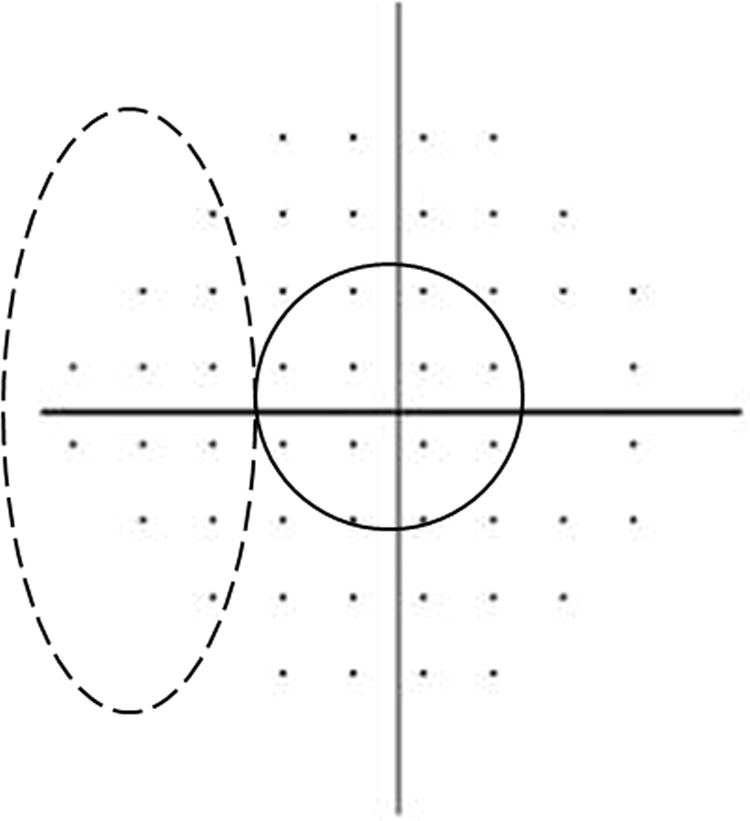
Pattern deviation plot divided into two subfields of the Humphrey visual field. The parafoveal scotoma group included abnormal points within 12 points of a central 10° radius (dashed line). The peripheral nasal step group had abnormal points within 12 nasal peripheral points (dotted line) in one hemifield.

The PFS and the PNS groups were determined by a single glaucoma specialist (K.I.J.) based on pattern deviation probability plots obtained from the SITA 24-2 test. PFS subjects had an isolated glaucomatous VF defects within twelve points of a central 10° radius in one hemifield (Fig. 1). PNS subjects had an isolated glaucomatous VF damage within the nasal periphery outside 10° of fixation in one hemifield. Subjects with VF defects in both the central 10° and the peripheral nasal fields, with VF defects other than the central or nasal periphery, or with scotoma in both the superior and inferior hemifields, were excluded from analysis. If glaucoma patients with PFS had VF defects in both the central 10° and peripheral nasal fields, they were assigned to the combined VF defect group. ERG data from combined VF defect group was additionally analyzed, even though the main purpose of this study was to compare the PFS and PNS group.

#### Electroretinography

ERG was performed using a commercial ERG stimulator (Neuro- ERG, Neurosoft, Ivanovo, Russia), a device that complies with the standards set by the International Society of Clinical Electrophysiology and Vision (ISCEV). A total of 4 electrodes were applied for stimulation. Two 35 mm Ag/AgCl skin electrodes were taped to the lower lids, with two ground electrodes at both earlobes. Black and white checkerboards with a check size of 1.81° were presented on a 24 inch-monitor with 48° × 33° visual angle, and at a distance of 60 cm. Stimuli were modulated in counterphase at 4 Hz. The checkerboards had a mean luminance of 105 cd/m². Participants fixed their views at the center of the monitor, where a red-colored fixed point was placed. The PERG was measured as binocular recordings with an appropriate refractive correction provided through undilated pupils. Responses were band-pass filtered (1-50 Hz), sampled at 10,000 Hz. At least 100 readings were recorded and averaged. To investigate the reproducibility of the ERG parameters, test-retest variability was measured with 34 randomly selected measurements.

The P50 amplitude was estimated from the trough of N35 to the peak of P50. The N95 amplitude was defined as being from the peak of P50 to the trough of N95. The implicit time for N35, P50, and N95 was measured from the onset of checkerboard reversal to the peak of each component.

### Statistical analysis

Statistical calculations of the PERG amplitudes showed a 79.2% difference between PFS and PNS patients in this study. Test-retest variability was calculated using the intraclass correlation coefficient (ICC) from a two-way mixed-effect model. ICC scores ≥0.75, 0.40–0.75, ≤0.40 are indicated to be excellent, moderate, and poor, respectively^17^.

SPSS software (ver. 17.0; SPSS Inc., Chicago, IL) was used for statistical analyses. Differences between the PFS and PNS groups were analyzed by the Student’s *t* test for continuous parameters and by the chi-square test for categorical parameters. Correlations between RNFL or GCIPL thickness and PERG amplitudes or SAP MD or MS were evaluated based on Pearson correlation coefficients. In all analyses, *P* < 0.05 was taken to indicate statistical significance

## Results

Among a total of 79 subjects, 27 were included in the normal control group, 29 glaucoma patients were included in the PFS group, and 23 in the PNS group. There were no statistically significant differences in age, gender, spherical equivalent, intraocular pressure or axial length between groups (Table 1). The MD of SAP 24-2 was lower and PSD was higher in the PFS or PNS group than the control group (All *P* < .001). Among glaucoma patients, no significant difference was observed in the MD or PSD between the PFS or PNS groups (All P > 0.05). Red-free fundus photographs of representative glaucoma patients and control subjects were presented in Supplementary Fig. S1.

**Table 1:** Demographics of subjects.

Parameter	Control group (n = 27)	PFS group (n = 29)	PNS group (n = 23)	*P value*		
				Control vs PFS	Control vs PNS	PFS vs PNS
Age (years)	46.2 ± 13.7	51.8 ± 11.0	50.2 ± 11.3	1.000	0.278	0.613
Male/Female	10/17	10/19	8/15	1.000	1.000	1.000
CCT (µm)	540.7 ± 47.4	530.5 ± 32.8	541.5 ± 46.1	0.406	0.961	0.354
Spherical equivalent (diopter)	−2.4 ± 2.9	−2.0 ± 2.8	−2.6 ± 3.4	0.634	0.822	0.502
Axial length (mm)	24.7 ± 1.1	24.9 ± 1.3	25.7 ± 1.3	0.643	0.061	0.131
IOP (mmHg)	14.1 ± 3.4	14.0 ± 3.2	15.3 ± 2.8	0.895	0.179	0.107
SAP MD (dB)	−1.1 ± 1.0	−2.8 ± 1.7	−2.6 ± 1.7	<**0.001**	<**0.001**	0.727
SAP PSD (dB)	1.6 ± 0.3	4.3 ± 2.2	3.8 ± 1.6	<**0.001**	<**0.001**	0.348

There were no statistically significant differences in any parameters except SAP MD and PSD between groups. Among glaucoma patients, no significant difference was observed in the MD or PSD between the PFS or PNS groups.

CCT, Central corneal thickness; MD, mean deviation; IOP, intraocular pressure; PFS, parafoveal scotoma; PNS, peripheral nasal step; PSD, pattern standard deviation; SAP, standard automated perimetry.

Continuous variables are expressed as n, mean ± standard deviation.

Glaucoma patients with PFS or PNS were found to have a thinner average RNFL than the control group (All P < 0.001, Table 2). There was no difference in average RNFL thickness between the PFS and PNS groups (P = 0.503). Average, minimum, superior, inferonasal, inferior, inferotemporal, and superotemporal GCIPL were thinner in the PFS group than in the PNS or control groups (P < 0.05 for all). There was no significant difference in optic disc parameters between the PFS and PNS group (All P > 0.05).

**Table 2:** Comparison of circumpapillary retinal nerve fiber layer and ganglion cell-inner plexiform layer thickness.

Cirrus OCT		Control group	PFS group	PNS group	P value		
					Control vs PFS	Control vs PNS	PFS vs PNS
RNFL thickness (µm)	Average	92.4 ± 7.8	76.5 ± 10.9	78.3 ± 8.4	<**0.001**	<**0.001**	0.503
	Superior	114.6 ± 15.6	99.9 ± 17.1	94.9 ± 20.9	**0.002**	**0.001**	0.352
	Nasal	63.4 ± 11.2	62.5 ± 8.3	62.8 ± 9.5	0.739	0.843	0.904
	Inferior	118.7 ± 12.4	85.5 ± 20.0	86.3 ± 18.2	<**0.001**	<**0.001**	0.878
	Temporal	72.1 ± 11.3	56.4 ± 15.0	69.4 ± 13.8	<**0.001**	0.473	**0.002**
GCIPL thickness (µm)	Average	81.4 ± 4.5	66.9 ± 9.2	76.6 ± 8.5	<**0.001**	**0.035**	**0.001**
	Minimum	78.1 ± 6.0	50.8 ± 10.1	66.5 ± 10.1	<**0.001**	<**0.001**	<**0.001**
	Superior	81.4 ± 4.6	71.5 ± 16.1	83.7 ± 12.6	**0.013**	0.457	**0.006**
	Superonasal	82.7 ± 7.0	72.1 ± 16.8	82.3 ± 15.4	**0.013**	0.914	**0.036**
	Inferonasal	82.2 ± 5.4	68.1 ± 11.4	78.5 ± 10.2	<**0.001**	0.163	**0.002**
	Inferior	78.8 ± 5.1	59.7 ± 8.1	69.3 ± 9.3	<**0.001**	<**0.001**	<**0.001**
	Inferotemporal	81.7 ± 4.4	59.7 ± 7.9	68.5 ± 10.3	<**0.001**	<**0.001**	**0.002**
	Superotemporal	80.6 ± 4.4	69.9 ± 13.1	77.6 ± 8.0	**0.001**	0.155	**0.021**
Optic disc parameters	Disc area(mm^2^)	1.9 ± 0.3	1.9 ± 0.3	1.9 ± 0.3	0.984	0.903	0.882
	Rim area(mm^2^)	1.2 ± 0.3	0.9 ± 0.2	0.9 ± 0.2	<**0.001**	<**0.001**	1.000
	Average CDR	0.5 ± 0.1	0.7 ± 0.1	0.7 ± 0.1	<**0.001**	<**0.001**	0.110
	Vertical CDR	0.5 ± 0.1	0.7 ± 0.1	0.7 ± 0.1	<**0.001**	<**0.001**	0.195
	Cup volume (mm^3^)	0.1 ± 0.1	0.5 ± 0.3	0.3 ± 0.2	<**0.001**	**0.001**	0.077

Glaucoma patients with PFS or PNS were found to have a thinner average RNFL, GCIPL, and more glaucomatous optic disc parameters than the control group. There was no difference in average RNFL thickness or optic disc parameters between the PFS and PNS groups. Several GCIPL parameters were thinner in the PFS group than in the PNS group.

CDR, cup-to-disc ratio; GCIPL, Ganglion cell-inner plexiform layer; PFS, initial parafoveal scotoma; PNS, initial peripheral nasal step; RNFL, Retinal nerve fiber layer.

The ERG measurements were found to have excellent reproducibility for the N95 amplitude (ICC = 0.827 and 95% CI = 0.645–0.910; Table 3), while the reproducibility for the P50 amplitude was moderate (ICC = 0.705 and 95% CI = 0.476–0.844). ICC values were moderate for the implicit times of N35, P50, and N95.

**Table 3:** Reliability of electroretinogram parameters.

		ICC	95% CI	P value
Amplitude (µV)	**P50**	0.705	0.476~0.844	<**0.001**
	**N95**	0.827	0.645~0.915	<**0.001**
Implicit time (ms)	**N35**	0.454	−0.092~0.728	**0.043**
	**P50**	0.646	0.292~0.823	**0.002**
	**N95**	0.481	−0.040~.741	0.032

The ERG measurements were found to have excellent reproducibility for the N95 amplitude. The reproducibility for the P50 amplitude was moderate. ICC values were moderate for the implicit times of N35, P50, and N95.

**Figure 2:**
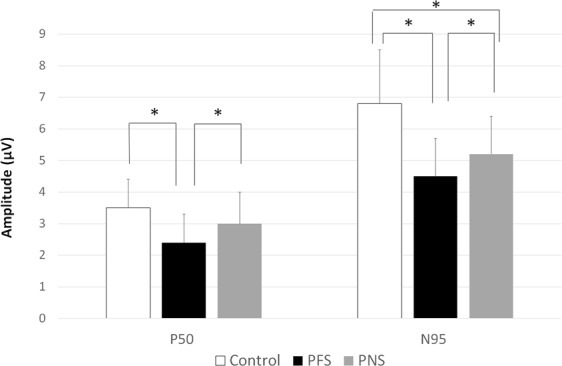
Comparison of the P50 and N95 pattern electroretinogram amplitudes in normal control subjects and glaucoma patients with parafoveal scotoma (PFS) or peripheral nasal step (PNS). *Statistically significant difference between groups, with P < 0.05.

A comparison of PERG parameters is shown in Table 4 and Fig. 2. The N95 amplitude was lower in both glaucoma groups than it was in the control group (P < 0.001 for both). The PFS group had a significantly decreased N95 amplitude than the PNS group (P = 0.034). Similarly, the P50 amplitude was reduced in the PFS group when compared to the control or PNS groups (P < 0.001, P = 0.023, respectively). The implicit time for the N35, P50, and N95 amplitudes did not differ between the groups (P > 0.05 for all). A multivariate analysis adjusted for age, sex, and average RNFL thickness showed a significant difference in N95 amplitude between the PFS and PNS groups (Beta coefficient, −0.706, 95% CI, −1.365~−0.046; P = 0.037). Glaucoma patients with both PFS and PNS displayed lower P50 and N95 amplitude than the control or PNS group (All P < 0.05, Supplementary Table S1). N95 amplitude was smaller in the combined VF defect group with both PFS and PNS than the PFS group (P = 0.008).

**Table 4:** Comparison of pattern electroretinogram parameters.

Pattern electroretinogram		Control group	PFS group	PNS group	*P value*		
					Control vs PFS	Control vs PNS	PFS vs PNS
Amplitude (µV)	**P50**	3.5 ± 0.9	2.4 ± 0.9	3.0 ± 1.0	<**0.001**	0.066	**0.023**
	**N95**	6.8 ± 1.7	4.5 ± 1.2	5.2 ± 1.2	<**0.001**	<**0.001**	**0.034**
Implicit time (ms)	**N35**	24.2 ± 2.9	24.9 ± 3.8	24.6 ± 3.6	0.420	0.642	0.770
	**P50**	49.9 ± 3.3	49.9 ± 3.5	50.7 ± 3.6	0.975	0.425	0.417
	**N95**	99.2 ± 7.1	101.2 ± 8.9	103.4 ± 8.3	0.358	0.060	0.366

Both PFS and PNS glaucoma groups showed lower N95 amplitude than the control group. The PFS group had a significantly decreased N95 and P50 amplitude than the PNS group. The implicit time for the N35, P50, and N95 amplitudes did not differ between the groups.

PFS, parafoveal scotoma; PNS, peripheral nasal step.

**Figure 3:**
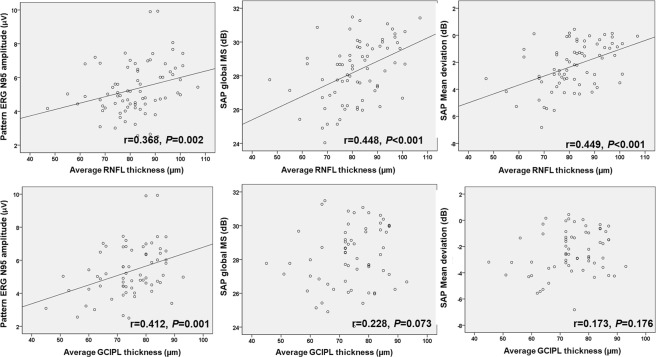
Scatterplots showing the relationship between average retinal nerve fiber layer (RNFL) thickness or average ganglion cell-inner plexiform layer (GCIPL) thickness and pattern electroretinogram (PERG) N95 amplitude or standard automated perimetry (SAP) mean sensitivity (MS) or SAP mean deviation.

Average RNFL thickness was positively correlated with N95 amplitude (r = 0.368, P = 0.002) and the global SAP MS (r = 0.448, P < 0.001), MD on SAP (r = 0.449, P < 0.001, Fig. 3). Average GCIPL thickness was also positively correlated with N95 amplitude (r = 0.412, P = 0.001), but did not correlate significantly with the global SAP MS (r = 0.228, P = 0.073) or MD on SAP (r = 0.173, P = 0.176).

P50 amplitude did not correlate with RNFL thickness, but did positively correlate with average, minimum, superonasal, inferonasal, inferior, inferotemporal, and superotemporal GCIPL thickness (0.269 < r < 0.472, P < 0.030; Table 5).

**Table 5:** Relationship between P50 or N95 amplitude and retinal nerve fiber layer (RNFL) or Ganglion cell-inner plexiform layer (GCIPL) thickness.

		P50 amplitude		N95 amplitude	
		r	P	r	P
RNFL thickness	Average	0.082	0.482	**0.368**	**0.002**
	Superior	0.039	0.739	0.193	0.098
	Nasal	−0.173	0.138	−0.049	0.201
	Inferior	0.136	0.245	**0.342**	**0.003**
	Temporal	0.170	0.144	**0.252**	**0.029**
GCIPL thickness	Average	**0.412**	**0.001**	**0.368**	**0.002**
	Minimum	**0.472**	<**0.001**	**0.439**	<**0.001**
	Superior	0.181	0.145	0.177	0.156
	Superonasal	**0.313**	**0.010**	**0.264**	**0.032**
	Inferonasal	**0.412**	**0.001**	**0.346**	**0.004**
	Inferior	**0.436**	<**0.001**	**0.381**	**0.002**
	Inferotemporal	**0.399**	**0.001**	**0.370**	**0.002**
	Superotemporal	**0.269**	**0.029**	**0.251**	**0.042**

Average RNFL and GCIPL thickness was positively correlated with N95 amplitude.

P50 amplitude did not correlate with RNFL thickness parameters, but did positively correlate with average and several sectorial GCIPL thickness.

r = Pearson’s correlation coefficient.

GCIPL, Ganglion cell-inner plexiform layer; RNFL, Retinal nerve fiber layer.

**Figure 4:**
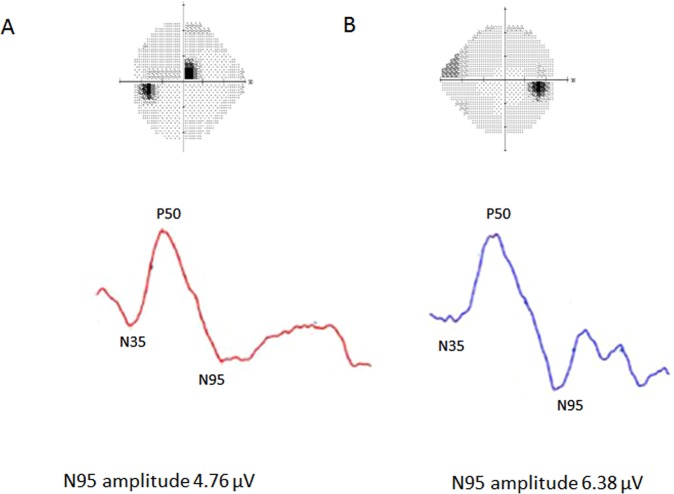
Representative cases with parafoveal scotoma (PFS) or peripheral nasal step (PNS). (**A**) A 58-year-old-woman with PFS: mean deviation (MD), −1.34 dB; pattern standard deviation (PSD), 6.37 dB; N95 amplitude 4.76 µV; P50 amplitude, 3.46 µV (**B**) A 58-year-old-woman with PNS: mean deviation (MD), −2.21 dB; pattern standard deviation (PSD), 2.15 dB; N95 amplitude, 6.38 µV; P50 amplitude, 3.56 µV.

Representative cases with parafoveal scotoma (PFS) or peripheral nasal step (PNS) were displayed in Fig. 4 (A: PFS, B: PNS). In two cases, MD was −1.34 dB in the A case and −2.21 dB in the B case. The N95 amplitude was lower in the patient with PFS (4.76 µV) than the patient with PNS (6.38 µV).

## Discussion

We demonstrated that the PERG amplitudes of glaucoma patients with PFS or PNS were lower than those of normal control subjects. Among glaucoma patients, the PFS group had a lower N95 PERG amplitude than the PNS group, even though both groups had a similar MD and PSD. Average GCIPL thickness was related to PERG N95 amplitude, but not to global SAP 24-2 MS or MD.

The PERG is known to measure the function of centrally located ganglion cells because it stimulates the 10~15° visual angle. Some PERG devices, such as the one used in this study, stimulate an extended visual angle of 44~48°^5,10^. Our study has revealed that the N95 PERG amplitude was lower in the PFS group than in the PNS group. Various estimates can account for this observed difference in N95 PERG amplitudes. First, there is a possibility that glaucoma patients with PFS lose more RGCs than patients with PNS do, even though the MDs and PSDs of the PFS and the PNS groups were not significantly different. The RGCs are disproportionately distributed and are more densely populated in the paracentral retina than in the peripheral retina^11^. Only 12 test points of the SAP 24-2 test fall within the central 10° VF, where more than 30% of RGCs are located^12^. Glaucomatous damage can be underestimated with SAP 24-2, especially in the macular area. In contrast, PERG may display the overall response from total RGCs within the stimulus. Relatively lower PERG N95 amplitude in the PFS group might reflect actual and more substantial RGC loss compared to the PNS group. It is our assumption and needs to be further elucidated. Second, the PERG response of central regions is known to be higher compared to eccentric regions even at their respective optimal spatial frequencies^14^. Damage of RGCs in the central region can contribute the substantial loss of PERG response. Therefore, the PFS group might show greater loss of PERG response because of the RGC loss in more central regions. The finding that the PFS group had a lower N95 PERG amplitude than the PNS group does not indicate that PERG amplitudes are able to discriminate between the patients with different types of VF defect such as PFS and PNS. We only found that parafoveal scotoma was associated with lower N95 PERG amplitude. We just recommend that we need to consider the location of VF defects in evaluating the PERGs of glaucoma patients.

Average, inferior, and temporal RNFL thickness positively correlated with N95 amplitude (Fig. 3). This corresponded with a previous study, which showed a positive correlation between average or inferior RNFL thickness and PERG amplitude in patients with early stages of glaucoma^18^. Several other studies have also reported a relationship between overall RNFL thickness and N95 amplitude^19,20^. Average RNFL thickness was also found to have a relationship with global SAP MS and MD (Fig. 3).

Average GCIPL thickness correlated positively with the P50 and N95 amplitudes, but no significant relationship was established with global SAP MD or MS (Fig. 3). These results are in general agreement with an earlier report by Park *et al*., who did not find a connection between macular GCIPL thickness and VF loss^9^. In that study, a significant association between GCIPL thickness and VF damage was found only in patients with advanced glaucoma^9^. In the present report, patients with early stage glaucoma were included: Mean MD was determined to be −2.8 dB in the PFS group and −2.6 dB in the PNS group. PERG amplitude seemed to reflect functional glaucomatous damage on the macula better than SAP 24-2 in early-stage glaucoma.

The P50 relates to the spatial distribution and density of the underlying retinal ganglion cell bodies^21^. Bach *et al*. reported that ganglion cell body could be the source of P50 and optic nerve head could be the origin of N95^21^. That could be the reason for the finding that P50 amplitude had a significant correlation with GCIPL thickness parameters but not with RNFL thickness.

Among ERG parameters, reproducibility was excellent for the N95 amplitude (ICC = 0.827). It was higher than the ICC (0.791) determined by Fredette *et al*., and lower than the ICC (0.91) reported by Bowd and colleagues^22,23^. This difference may result from the use of different instruments or glaucoma severity. The reproducibility of implicit time was relatively lower than that of amplitude parameters.

Recording PERGs is challenging due to the use of invasive corneal contact electrodes, and the difficulty of examination hampers widespread use of PERGs. Pattern stimulus visibility may be unclear with corneal electrodes attached to a contact lens or speculum. In this study, we employed a noninvasive method by using skin electrodes tapered on the lower eyelids with a commercially available PERG instrument. When compared in several other studies, PERG measurements obtained from electrodes on the skin were comparable to those obtained from corneal electrodes^24,25^.

One limitation of this study is the relatively small sample size in each group. As far as we know, however, this is the first study to evaluate the relationship between VF location and PERG with extended visual angle. Larger and more carefully controlled prospective studies are needed to confirm the results obtained here. Our results cannot be applied to the conventional PERG with the narrower visual angle which does not cover the peripheral VF. Another limitation is that the 48° horizontal visual angle of PERG is smaller than the 54° horizontal visual angle of SAP 24-2, because SAP 24-2 examines visual field from 30° nasally. Visual function measured using PERG may be underestimated in patients with PNS than those with PFS. However, the difference between the PERG and SAP 24-2 horizontal visual angles is very small.

In summary, we found that the location of VF defects in glaucoma patients affected the PERG amplitude. Lack of familiarity with PERG hampers its widespread use in clinical practice, despite its great potential as an objective assessment. Our results will increase the general understanding of the nature of PERG, indicating that there is a need to consider the location of VF defects in evaluating the PERGs of glaucoma patients. In addition, our investigation of visual function in the paracentral retinal region is significant because of the clinical importance of central visual function. GCIPL thickness was positively related to PERG amplitude, but not to global SAP parameters. Therefore, PERG, in addition to SAP 24-2, may be clinically helpful in the functional evaluation of early glaucoma patients with paracentral scotoma.

## Supplementary information

Supplementary file
