# Supplementary material for: Comparison of pattern electroretinograms of glaucoma patients with parafoveal scotoma versus peripheral nasal step
Source: Sci Rep. 2019 Mar 5;9:3547. doi: 10.1038/s41598-019-39948-y (PMC6401083; doi:10.1038/s41598-019-39948-y)
Supplement: Supplementary file 1 — Supplementary file [file 41598_2019_39948_MOESM1_ESM.pdf]

Comparison of pattern electroretinograms of glaucoma patients with parafoveal scotoma versus peripheral nasal step

Kyoung In Jung, M.D., Ph.D., Sooji Jeon, M.D., Yong Chan Kim, M.D., Chan Kee Park, M.D., Ph.D

-Supplementary files-

Supplementary figure S1.

Supplementary table S1

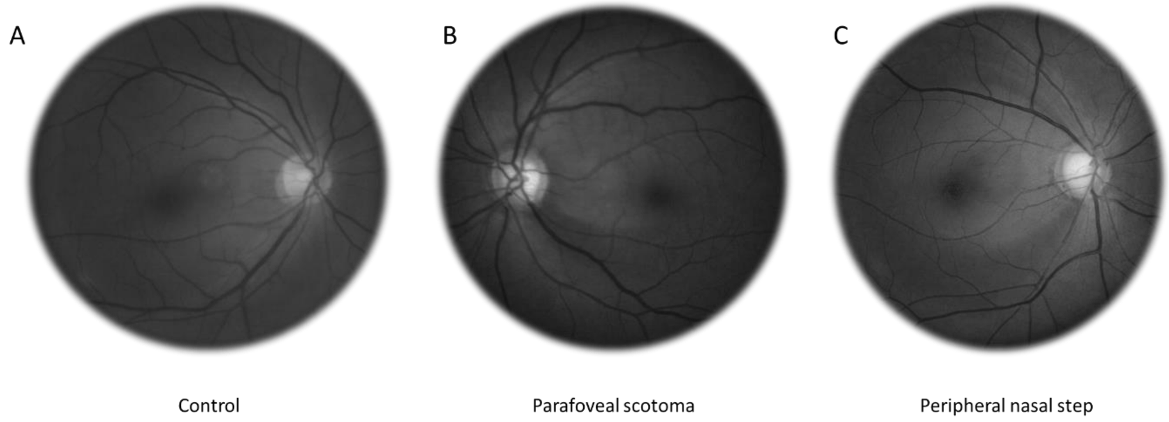

Supplementary Fig.S1 Representative red-free fundus photographs of control subjects (A), glaucoma patients with parafoveal scotoma (B), and those with peripheral nasal step (C)

Supplementary table S1. Visual field and pattern electroretinogram parameters in combined visual field defect group

Parameters			Control group (n=27)	IPFS group (n =29)	IPNS group (n =23)	Combined VF defect group (n=25)	*P value*		
							**Control vs Combined VF defect group**	**IPNS vs Combined VF defect group**	**IPFS vs Combined VF defect group**
**SAP**	**MD (dB)**		**-1.1±1.0**	**-2.8±1.7**	**-2.6±1.7**	**-14.7±4.8**	**<0.001**	**<0.001**	**<0.001**
	**PSD (dB)**		**1.6±0.3**	**4.3±2.2**	**3.8±1.6**	**11.6±2.1**	**<0.001**	**<0.001**	**<0.001**
**Pattern electroretinogram**	**Amplitude (μV)**	**P50**	**3.5±0.9**	**2.4±0.9**	**3.0±1.0**	**2.2±1.1**	**<0.001**	**0.008**	0.422
		**N95**	**6.8±1.7**	**4.5±1.2**	**5.2±1.2**	**3.5±1.4**	**<0.001**	**<0.001**	**0.008**
	**Implicit time (ms)**	**N35**	**24.2±2.9**	**24.9±3.8**	**24.6±3.6**	**25.0±3.7**	0.940	0.770	0.719
		**P50**	**49.9±3.3**	**49.9±3.5**	**50.7±3.6**	**49.4±3.4**	0.618	0.417	0.208
		**N95**	**99.2±7.1**	**101.2±8.9**	**103.4±8.3**	**99.6±7.2**	0.475	0.366	0.096

MD, mean deviation; IPFS, initial parafoveal scotoma; IPNS, initial peripheral nasal step; PSD, pattern standard deviation; SAP, standard automated perimetry
